# The Assessment of Prolonged Inferior Alveolar Nerve Blockade for Postoperative Analgesia in Mandibular Third Molar Surgery by a Perineural Addition of Dexamethasone to 0.5% Ropivacaine: A Randomized Comparison Study

**DOI:** 10.3390/ijerph19031324

**Published:** 2022-01-25

**Authors:** Simona Stojanović, Nikola Burić, Milos Tijanić, Kosta Todorović, Kristina Burić, Nina Burić, Marija Jovanović, Vukadin Bajagić

**Affiliations:** 1 Department of Oral Surgery, School of Medicine and Stomatology, University of Niš, 18101 Niš, Serbia; tarana.simona@gmail.com (S.S.); tijanicm@yahoo.com (M.T.); kosta_todorovic@yahoo.com (K.T.); 2 School of Medicine, University of Niš, 18101 Niš, Serbia; kristinaburic@yahoo.com (K.B.); ninaburic@yahoo.com (N.B.); prof.g.jovanovic@gmail.com (M.J.); 3 School of Medicine, University of Podgorica, 81110 Podgorica, Montenegro; highlandernsv@yahoo.com

**Keywords:** ropivacaine, dexamethasone, prolonged anaesthesia, postoperative analgesia, mandibular anesthesia

## Abstract

*Background:* Perineurally adding dexamethasone to local anesthetics could enable postoperative analgesia. Our aim was to investigate the efficacy of 4 mg dexamethasone and 0.5% ropivacaine on the prolonged duration of mandibular anesthesia for postoperative analgesia during third molar surgery. *Materials and method:* The patients of both sexes, and in the age range of 17 to 50 yrs of age, received the Gow-Gates anesthesia. Group I received 4 mL of plain 0.5% ropivacaine, with perineurally added 1 mL/4 mg of dexamethasone; group II received 4 mL of plain 0.5% ropivacaine with perineurally added 1 mL of 0.9% saline; group III received 4 mL of plain 0.5 bupivacaine with perineurally added 1 mL of 0.9% saline. The prime anesthesia outcome was the duration of conduction anesthesia (DCA); the secondary outcome was the duration of analgesia (DAN) and analgesia before analgesic intake. *Results:* In 45 randomly selected subjects (mean age 27.06 ± 8.20), DCA was statistically longest in group I (*n* = 15) (592.50 ± 161.75 min, *p* = 0.001), collated with groups II (*n* = 15) and III (*n* = 15) (307.40 ± 84.71 and 367.07 ± 170.52 min, respectively). DAN was significantly the longest in group I (mean: 654.9 ± 198.4 min, *p* = 0.001), compared with group II (345.4 ± 88.0 min) and group III (413.7 ± 152.3 min), with insignificant adverse reactions. One-third of the operated patients absented from the use of analgesics. *Conclusion:* A amount 0.5% ropivacaine with dexamethasone usefully served as an analgesic with a success rate of 93.4% of the given anesthesia.

## 1. Introduction

Pain is the most frequent and most experienced discomfort after oral surgery, especially after lower third molar surgery [1], which is performed in everyday oral and maxillofacial ambulatory praxis [2]. This unpleasant clinical outcome, which is the consequence of surgery, is mainly provoked by the pathological influence of the released mediators of pain (histamine, serotonin, kinin and prostaglandins), triggered by the locally surgically damaged dentoalveolar tissue [3], occurring up to 5 h after surgery upon the cessation of the local anesthetic’s efficacy [4]. The devastating fact is that severe pain is experienced by up to 93% of patients in the first postoperative 24 h [5], with a peak in pain intensity 3–12 h after surgery [4,6,7,8]. The use of ropivacaine, an amide long-lasting local anesthetic in dentistry and oral and maxillofacial surgery is not quite common, and the first reported case of ropivacaine use is described in our country for maxillary sinus surgery [9]. Ropivacaine is also used as a long-lasting amid anesthetic in various types of oral surgery practice but not only for impacted third molar surgery [10]. Some studies show that the patients could be relieved of the first postoperative pain to a certain extent when compared to those who received plain 0.75% ropivacaine and to 0.5% bupivacaine, but analgesics and anti-inflammatories are almost mandatory during this time [11]. Considering the possible toxic reaction of cardiovascular and neural tissue, ropivacaine has been shown to be a safer anesthetic than bupivacaine [12,13].

It is important evidence that the postsurgical pain, which is acute pain, can last not only for 24 h immediately after surgery but up to several days [14] and could be categorized as moderate, severe or extreme pain [15], which can lead to significant physiological, emotional, mental and economic consequences to the patient [16].

Therefore, pain management in clinical surgical praxis constantly exhibits the need for a pain-free early postoperative period of the surgical patient for as long as possible, achieved in the safest, simplest and most practical clinical manner. It is not surprising that, for that purpose, there is evidence of the use of long-acting local anesthetics for achieving longer-lasting intraoperative local anesthesia in oral surgery [10,11] and controlling postoperative pain, thus increasing the early postoperative pain-free interval and minimizing the intake of postoperative analgesic [1,17]. In addition, adding different adjuvants to local anesthetics for the purpose of prolonging the effects of local anesthesia for clinically relevant postoperative analgesia is described in surgical practice [18,19]. Some additional drugs, such as clonidine, pethidine, morphine, butorphanol and midazolam, are added to local anesthetics for more intense, faster and longer-lasting local anesthetics [20,21,22], followed by neostigmine and α2-agonists [23,24,25] and ketamine, but with unsatisfactory overall results [26]. It is shown that the everyday practical use of some of the cited drugs (morphine, pethidine and butorphanol) in ambulatory surgery is not practical and is difficult, owing to their unfavorable pharmacological side-effects, such as respiratory depression, severe sedation and psychotomimetic effects [27].

It is also an even greater challenge for the clinician to achieve the prolonged duration of postoperative anesthesia for the purpose of realizing postoperative analgesia with one single-shot of local anesthetics for the inferior alveolar nerve block (IANB). There have been numerous medical attempts to achieve the prolonged effect of local anesthetic nerve blockade by increasing the dose of local anesthetics or by developing new drugs for that purpose, but all these attempts have been unsuccessful, mainly because of the indicated toxic episodes [28,29].

It is a known clinical fact that dexamethasone is commonly used perioperatively for controlling and minimizing postoperative pain, nausea and vomiting, enabling a better patient recovery [30,31]. Literature data suggest that corticosteroids show the effectiveness in reducing postoperative pain in oral, general and orthopedic surgery [32,33]. As a selective glucocorticoid, dexamethasone exhibits high pharmacodynamic potency, which is about 40 times that of hydrocortisone; dexamethasone serves for the treatment of various autoimmune and inflammatory diseases [18,34]. The clinical uses of dexamethasone are chiefly for the treatment of various inflammatory and autoimmune conditions [34]. The use of dexamethasone for prolonging the duration of local/regional anesthesia has shown promising results in general surgery, confirming that 8 mg of dexamethasone, perineurally added to local anesthetics, prolongs the duration of the peripheral nerve block analgesia after supraclavicular brachial plexus and interscalene nerve blockades [35,36]. Dexamethasone acts by a direct blockade of the pain stimuli and reduces inflammatory mediators with a subsequent upregulation of potassium channels [37]. Pedersen used decadron phosphate in his study, and gained pain reduction after the removal of the impacted mandibular molars [38]. Although there is a concern about the potential neurotoxicity of perineurally applied dexamethasone [39], the research data did not confirm this [40].

Different routes for administering dexamethasone are described in oral surgical practice as the submucosal route of the injection of dexamethasone near the operated mandibular wisdom tooth in controlling post-operative sequelae [41,42,43]. Preoperative injection of dexamethasone into the masseter muscle [38,44] showed a significant reduction in pain, swelling and trismus after third molar surgery.

To date, to the best of our knowledge, we have found a lack of oral and maxillofacial literature data reporting on the beneficial effects of dexamethasone, in combination with ropivacaine, on prolonged anesthesia and enabling sufficiently long postoperative analgesia for the purpose of enabling the patient to experience a postoperative pain-free period without analgesic medication intake; also in addition, it would be desirable to achieve profound IANB anesthesia with a high success rate.

We have hypothesized that administered dexamethasone, together with a long-lasting local anesthetic—plain 0.5% ropivacaine, injected simultaneously perineurally in the pterygomandibular space, provides a significant prolongation in the duration of mandibular conduction anesthesia (primary outcome), with subsequent prolongation the duration of postoperative analgesia (secondary outcome).

Thus, the aim of this study is to investigate whether the patients undergoing mandibular third molar surgery receiving intra-spaced simultaneous perineural injections of 4 mg dexamethasone and plain 0.5% ropivacaine during the course of conduction mandibular anesthesia exhibit a profound and prolonged duration of mandibular conduction anesthesia, which serves as an analgesic for sufficient postoperative analgesia.

## 2. Materials and Methods

### 2.1. Subject Selection

This randomized double blind clinical study has been approved by our Institutional Ethical Board (# 12-7476-2/2, dated 1 July 2019), and the cohort of the investigated patients gave their informed written consent to the performed procedures after being given oral and written explanations of this study.

CONSORT (Consolidate Standards of Reporting Trials) principles were applied in this study, and the selection of all the included arbitrarily selected patients was performed by using the sealed envelope technique, previously used in similar studies [11]. Out-patients of both sexes, in the age range of 17 to 50, with physical status Grade I (ASA I), were selected for this study, according to the American Society of Anesthesiologists.

#### 2.1.1. Study Design

The inclusion criteria included a randomly selected asymptomatic cohort with radiologically classified horizontally impacted third molars, according to the Winter’ s scale of mandibular third molar impaction [11], and without pain, trismus, swelling, infection, pericoronitis, corticosteroid medications and antibiotic therapy 14 days before surgery.

#### 2.1.2. Exclusion Criteria

Exclusion criteria were applied for unwilling patients for this study, and the patients with a history of immunocompromised conditions, as well as those with medical conditions which could compromise this study, such as allergies to food or drugs, corticosteroid used for at least 2 weeks or longer before this investigation, endocrine disorders, peptic ulcer disease, pregnancy, breast feeding condition, malignant diseases and subsequent antineoplastic therapy.

### 2.2. Techniques of Injections of Local Anesthetics and Dexamethasone

Similar disposable sterile plastic 5 mL syringes (Nipro syringe, Shanghai International Holding Corp.GmbH /Europe, Eifestrasse 80, 20537 Hamburg, Germany), serving as containers for local anesthetics (with proofing the expiration date), were used for all groups. With regard to the sterility of the needle, a 21 G × 1½“ 0.8 × 40 mm needle (Nipro needle, Nipro Europe N.V., Weihoek 3H,B-1930 Zaventem, Belgium) was used for the injection of the testing drug to the target point. The following local anesthetics were used for conduction mandibular anesthesia: 4 mL of plain 0.5% ropivacaine (ROPIvacain 5 mg/mL, B|Braun Melsungen AG, 34209 Melsungen, Deutschland), with perineurally added 1 mL/4 mg of dexamethasone (Dexason^®^ 4 mg, GALENIKA AD BEOGRAD, Batajnički drum b.b., Beograd, Republika Srbija); 4 mL of plain 0.5% ropivacaine (ROPIvacain 5 mg/mL, B|Braun Melsungen AG, 34209 Melsungen, Deutschland) with an added 1 mL of sterile 0.9% saline; and 4 mL of plain 0.5% bupivacaine (Marcaine^®^, 0.5% Astra Zeneca, Sodertalje, Sweden) with an added 1 mL of sterile 0.9% saline. 

Mandibular conduction anesthesia was given to all groups in an identical manner, according to the technique by Gow-Gates [45], with the target point of the needle’s tip to the condylar neck at the beginning of the sulcus coli mandibule and with the mandatory additional aspiration maneuver before the deposition of the whole solution. After the completed injection of the selected local anesthetic fluid, the syringe is disconnected from the needle, firmly holding the needle “in position” with an 18 cm long curve Rochester pean forceps. Then, a new syringe with 1 mL of dexamethasone or sterile 0.9% saline is reconnected to the needle “in situ”. The needle was then pulled out by 3–5 mm to the medial line of the oral cavity and redirected caudally towards the inner side of the mandible until the contact with the perilingula’s mandibular bone was achieved. The subsequent maneuver involved pulling out the new syringe by 1 mm, with another mandatory aspiration test, and the final deposition of dexamethasone or sterile 0.9% saline, dependent on the investigated group, performed perineurally.

In cases of the insufficient effectiveness of local anesthesia before surgery, which is manifested in patient’s painful reaction to the stabbing of the lip and mandibular vestibular mucosa with blunt instruments (dental surgical tweezers pincette), on the ipsilateral (operated) side of mandible, an additional 2 mL of the same plain local anesthetic was repeated, dependent on the investigated group and injected according to the Gow-Gates technique, with additional notification in investigation paper [11]. Otherwise, in case that patient showed negative reaction on lip and mucosal stabbing, the surgery of wisdom tooth starts because effective anesthesia is considered to have been achieved.

### 2.3. Wisdom Tooth Surgery

The surgery of a patient’s impacted mandibular wisdom tooth was performed with the same standard surgical technique for all patients. Preoperatively, for extraoral antisepsis, an antiseptic solution of 10% iodine was used for gentle skin scrubbing in all patients. This lasted for 3 min and was followed by intraoral antisepsis with 20 mL of 0.12% chlorhexidine gluconate, also lasting 3 min. Access to the impacted third molar was performed with a linear incision, using scalpel # 15, by means of a buccal approach through the gingival sulcus, starting from the interdental papillae of the first molar, intrasulcularly extended distally and laterally from the second molar and, projecting to the anterior ramus margin with a vertical incision (5 mm long), with a subsequent raising of the mucoperiosteal envelope flap. The impacted wisdom tooth was freed by bone removal around the crown of the tooth with the use of a sterile round bur (# 167-141, Meisinger HM, Neuss, Germany) fixed on a surgically straight hand-piece and with the copious irrigation of cold (8 °C) and sterile 0.9% physiological saline (sodium chloride); odontectomy of the crown was performed from the roots at the cervical level and the roots themselves. Post-extraction, the surgical wound was irrigated and cleansed from any debris, while the envelope flap was repositioned carefully to match the previous anatomical position and sutured through the dental papilla and mucosal incision by means of a 5–0 polyglicolic acid suture (Marline rapide USP 5/0, Catgut GmbH, Gewerbepark 18, 08258 Markneukirchen, Germany), with subsequent suture removal time on the seventh postoperative day. Antibiotic therapy was administered orally, preferably by oral route: amoxicillin 875 mg, with 125 mg of clavulanic acid (Augmentin 1000, SmithKline Beecham Pharmaceuticals Clarendon Road, Worthing, West Sussex BN14 8QH, Great Britain), 2 × 1 g/day for 7 days and, in case of a possible allergy to a penicillin derivate, clindamycin therapy was introduced (Clindamycin MIP 600, St Ingberg, Germany), 2–3 × 600 mg every 8–12 h per day, 7 days in duration. For the elimination of pain, the patients were instructed to use a non-steroid anti-inflammatory drug (Brufen 400, Galenika, Belgrade, Serbia), 400 mg twice a day, and to report that they were taking it. Patients were asked to monitor and record the first pain time and the time when analgesics were taken with records of pain intensity on an NRS scale. A recalled session for the final removal of sutures was scheduled upon the seventh postoperative day, and after that, further observation of the patients was ended.

### 2.4. Summary of Anesthesia and Analgesia Parameters with Defined Outcomes

Some objective and subjective measures were taken in order to measure the anesthetic efficiency of conduction anesthesia of the tested long-acting local anesthetics in the elimination of intraoperative pain and prolong postoperative anesthesia for postoperative analgesia. 

The primary outcome in this study is the duration of conduction anesthesia (DCA), considered as the time from the first sign of the patient’s lip numbness to the moment when lip numbness disappeared.

**Figure 1:**
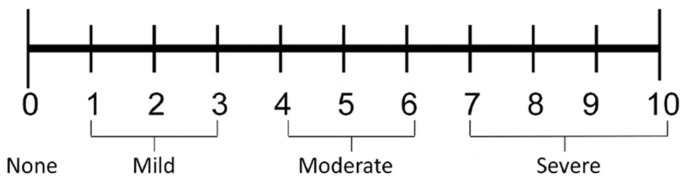
The used numerical rating scale (Mc Caffery et al., 1989).

The anesthetic efficacy parameter was measured by using Sisk’s scale [46], which defines the quality of the anesthesia score (QAS) that objectively and practically measures anesthesia effectiveness during surgery with the following parameters: 1. Successful—no pain throughout the procedure; 2. Successful—some pain during the procedure, but reinjection was not necessary after the beginning of the surgery; 3. Successful—pain during the procedure, beginning after the first injection, and no pain after the second injection; 4. Limited success—pain during the procedure, beginning after the first injection; pain also during the procedure, after the second injection, but surgery completed without the third injection; 5. Limited success—pain during the procedure, beginning after two injections, but surgery completed without the third injection; 6. Failure—pain during the procedure, beginning after the first injection, pain also during the procedure after the second injection, third injection required; 7. Failure—pain during the procedure, beginning after two injections, third injection required; 8. Failure—no anesthesia after two injections, third injection required or treatment suspended. The evaluation of the effectiveness (painlessness) of the applied local anesthesia solutions for conduction mandibular anesthesia and postoperative analgesia was performed by the patients (respondents) selected for this study, and statistically processed [47]. All the respondents measured the experienced intraoperative/postoperative pain level perception during the impacted tooth mandibular surgeries and, postoperatively, the analgesic effects of the applied local anesthesia by using the one-dimensional, numerical rating pain intensity scale (NRS) acc. to Downie et al. [48] and Flaherty [49]. The used NRS is presented as a horizontal straight, 11-point scale [50]. When using the NRS, patients were asked to rate the severity of their pain on a scale from 0 to 10 by simply circling the whole number (0–10 integers), 0 representing “no pain”, 1–3 representing “mild pain”, 4–6 describing “moderate pain” and 7–10 representing “severe pain”, which was used in other similar studies [51], as with the verbal rating scale [52], (Figure 1).

The overall success rate of local anesthesia effectiveness (without supplemental anesthesia) is also expressed in percentages.

The secondary outcomes included: minutely measured onset time (OT), which is defined as the time from the injection of local anesthesia to the target site to the moment of the patient’s lip numbness onset (lingual and buccal nerves’ altered sensation is included), with the negative probing of lingual and buccal nerves, as well; additional anesthesia(#); the quantity of the administered local anesthetics (QUA) in mL per patient; the # of patients used analgesics; perioperatively NRS; NRS of pain when the analgesics used; duration of analgesia (DAN) when analgesics used for the first time (min); amount of analgesics intaken 24 h postoperatively. The duration of analgesia is recorded from the moment of the completion of surgery to the moment of the first experienced postoperative pain and use of analgesics with its variables (analgesic intake, time of the first analgesic, # of analgesics).

Other secondary measured parameters included all local and systemic parameters related to the deposited local anesthetic and other medications, as well as adverse reactions, such as syncope (Sy), hematoma (HeM), syringe aspiration of blood (SAB), nausea (NaU), tinnitus (TiN), palpitations (PP), dizziness (DiZ), drowsiness (DrW) and other side effects, which were recorded.

### 2.5. Sample Size

The sample size encompasses 3 equal groups, with 15 participants per group, testing for the prolonged duration and quality of anesthesia for local anesthetic comparison (0.5% ropivacaine, with added 4 mg of dexamethasone, plain 0.5% ropivacaine and plain 0.5% bupivacaine) along with monitoring local anesthesia outcomes. The determination of the sample size was performed with the software package G power 3.1.9.2. (version 3.1.9.2) [47]. The initial parameters were defined for the study of 80%, and the probability of the first type of error (α) was 0.05 for two-way testing of the null hypothesis, proven in the previous research [36]. The estimated sample size is 15 subjects per group.

The posthoc power analysis was obtained through the G Power statistical computing program (version 3.1.9.2) [45] and confirmed that the sample size of 15 patients per group gives a minimum 80% level of the study power (0.80).

### 2.6. Statistical Analysis

The obtained results are statistically processed and analyzed with the statistical program using SPSS (version 15.0; SPSS, Chicago, IL, USA).

The primary endpoint data were analyzed with the Kruskal–Wallis test and Man–Whitney test (post hoc analysis) to compare the values between groups. The following secondary endpoints, onset of anesthesia, quantity of anesthesia per patient, duration of analgesia, duration of analgesia when the analgesics were used for the first time, amount of analgesics 24 h postoperatively, NRS of pain during surgery and NRS of pain when the first analgesics used were analyzed by Kruskal–Wallis test. The following secondary endpoints, additional anesthesia, complete success of LA achieved by one local anesthesia were analyzed by the chi-squared test or Fisher test (as appropriate). The data are presented in the form of an arithmetic mean ± standard deviation or as a count and percentages in the form of absolute and relative numbers. The significance threshold for the given hypothesis and for all the statistical comparisons was *p* value < 0.05.

## 3. Results

A total of 52 patients were randomly screened for this study. Of these, 7 patients did not meet the inclusion criteria and were excluded from further investigation, mainly due to technical difficulties during the surgery, or the patient’s refusal for further study, so the cohort of 15 subjects per group was formed and divided into 3 equal groups. The primary and secondary local anesthesia outcomes during lower impacted third molar surgery were registered in patients who had received plain 0.5% ropivacaine with the perineural adding of 4 mg dexamethasone (group I), plain 0.5% ropivacaine (group II) and plain 0.5% bupivacaine (group III). All three groups of subjects underwent the surgery of impacted lower third molars in the horizontal position; perioperatively, local anesthesia outcomes were recorded in all groups in an identical manner. The subjects’ confident interval (CI) was 95%; all the data are presented in Table 1.

**Figure 2:**
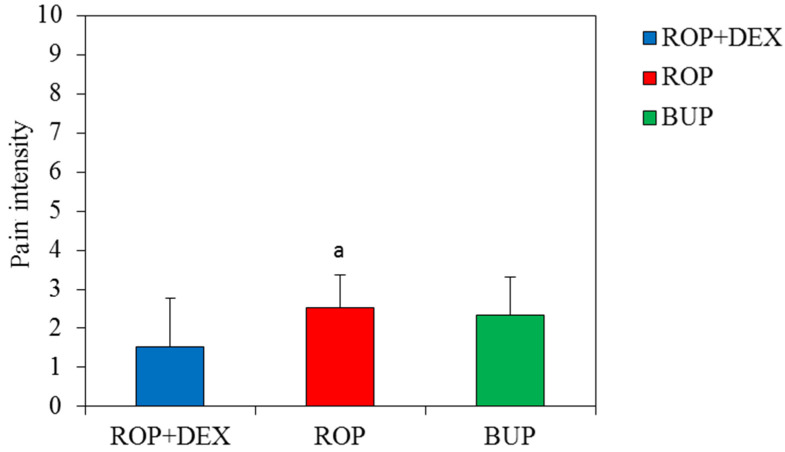
NRS assessment of pain during surgery. Abbreviations: rop + dex (plain 0.5% ropivacaine plus 1 mL dexamethasone, added perineurally), rop (plain 0.5% ropivacaine) and bup (0.5% plain bupivacaine).

The mean pain intensity of the NRS values during the surgery was higher in plain 0.5% ropivacaine and 0.5% bupivacaine groups (2.53 ± 0.83 and 2.33 ± 0.98, respectively), compared to the statistically lower values (1.53 ± 1.25, *p* = 0.044) of the pain intensity in 0.5 % ropivacaine and dexamethasone group (Figure 2).

There is a statistical difference between the tested groups considering the achieved duration of anesthesia (primary outcome) compared with the achieved analgesia (secondary outcome) (*p* = 0.001). In group I, the longest duration of local anesthesia was achieved (549.73 ± 224.56 min) compared to groups II and III (277.0 ± 61.52 and 316.47 ± 151.88 min, respectively). The overall results for the achieved anesthesia and analgesia are statistically significant in favor of group I when compared to the anesthesia and analgesia parameters of group II (*p* = 0.001 and *p* = 0.001, respectively), and the anesthesia and analgesia parameters of group III (*p* = 0.001 and *p* = 0.007, respectively) (Table 2).

The achieved secondary outcomes were as follows.

The onset time of conduction anesthesia for the IANB block was between 4 and 5 min in the tested groups, with the fastest mean onset results in group I (4.13 ± 1.30), followed by groups II and III (5.40 ± 2.10 and 4.47 ± 1.46, respectively) but without significance. The lowest number (1) of additional anesthesia was in group I. (Table 2).

The amount of the injected local anesthetics was almost the same in all three groups (between 4 and 5 mL). In 33.3% of the patients in group I, there was no use of analgesics, which is clinically significant. Almost the same amount of injected local anesthesia (4–5 mL) was administered in all three groups (Table 2). The significant lowest number of used analgesics 1.90 ± 0.57 (*p* = 0.027); the overall lowest number of patients that used analgesics for postoperative analgesia is in favor of group I. The lowest statistically significant value of NRS when the first analgesics used, was recorded in group I (3.70 ± 0.68, *p* = 0.004), with the longest period of postoperative analgesia observed when the first pain killer was used in group I (654.9 ± 198.4 min, *p* = 0.001). All the collected data are presented in Table 2.

The duration of analgesia was statistically the longest in group I (592.50 ± 161.75 min, *p* = 0.001), while significantly shorter analgesia was achieved in groups II and III (in the duration of 307.40 ± 84.71 and 367.07 ± 170.52 min., respectively). When the first analgesics was used, the mean duration of analgesia was 654.9 ± 198.4 min, 345.4 ± 88.0 min and 413.7 ± 152.3 min, for groups I, II and III, respectively, and this duration of analgesia in group I is statistically significant (*p* = 0.003) (Table 2), with the significant lowest number of used analgesics 1.90 ± 0.57 (*p* = 0.027); the overall lowest number of patients that used analgesics for postoperative analgesia is in favor of group I (Table 2).

The analysis of the success of local anesthesia in absolute terms (one injection of local anesthesia for a successful IANB block without supplemental local anesthesia) showed that success was achieved in 93.4% of the patients in group I (14), with supplemental anesthesia in one patient (6.7%); in group II, 66.7% of the patients (10) had successful anesthesia effects for the IANB block, with additional anesthesia in 5 patients (33.3%); in group III, 60% of the patients (9) exhibited successful IANB anesthesia, with additional anesthesia in 6 patients (40%) (Table 2).

The recorded results of complete success in absolute terms are statistically significant different for groups I and III, in favour of ropivacaine and dexamethasone group results (*p* < 0.05, *p* = 0.034, Mantel–Haenszel chi square test) (Table 2).

The administered long-acting local anesthetics for IANB block anesthesia showed that the QAS is statistically different among the tested groups, and the best statistically significant mean quality of anesthesia score is achieved with plain 0.5% ropivacaine, with perineurally added dexamethasone (group I) (1.47 ± 0.38, *p* = 0.037) (between the rates successful—without pain and successful—minimal pain during the procedure without additional anesthesia after the start of surgery) compared with plain 0.5% ropivacaine (2.40 ± 1.31, *p* = 0.023—post hoc analysis) (group II) and plain 0.5% bupivacaine (2.27 ± 1.00, *p* = 0.037—post hoc analysis) (group III) (between the rates: successful—minimal pain during the procedure without additional anesthesia after the start of surgery and successful—minimal pain after the first anesthesia, no pain after another anesthesia) (Table 3).

Other recorded secondary outcomes:

Dizziness occurred in groups II and III before anesthetic administration but no patients were injected with the tested anesthetics after this. One patient in group III had pronounced drowsiness lasting up to 2 days after surgery but without serious medical complications. The occurrence of two hematomas was noted with positive aspiration tests in the tested groups. The paresthesia of the lingual nerve was present in one patient but disappeared after 3 weeks (Table 2).

## 4. Discussion

The tested hypothesis in this paper was that a simultaneously added perineural injection of dexamethasone to long-lasting anesthetics has a significant impact on duration of anesthesia and successfully prolongs the effects of local anesthetic serving as analgesics.

Considering the quality of the prolonged intraoperative anesthesia and achieved postoperative analgesia, our study clearly demonstrates the effectiveness of the combination of perineurally added dexamethasone to the almost baseline concentration of plain 0.5% ropivacaine, with one intra-space shot into the pterygomandibular space during the course of IANB conduction anesthesia compared with plain 0.5% ropivacaine and 0.5% bupivacaine in wisdom tooth surgery. The duration of intraoperative anesthesia and gained postoperative analgesia for the pain relief period was markedly prolonged in ropivacaine plus dexamethasone group without side effects when compared with the other two groups. Our encouraging results about adding dexamethasone to long-lasting anesthetics are not a surprise because added dexamethasone to local anesthetics exhibits an improvement in pain reduction and prolonged postoperative anesthesia is successfully used in different surgical models, such as supraclavicular brachial plexus block, as reported [53], and in orofacial surgery for lesser morbidity of the third molar surgery [54,55,56], but with a different route from ours, considering corticosteroid administration.

The gained onset time of conduction anesthesia for the IANB block with the tested anesthetic solutions showed approximately the same results and was in the range of 4 and 5 min for all the tested groups, but the fastest mean onset results were recorded in group I (4.13 ± 1.30 min) compared to groups III and II, but without statistical significance; these results are in disagreement with the previous study of Tijanić and Burić [11], who reported a shorter onset time of plain 0.75% ropivacaine (≈2.5 min). The achieved faster onset time in the previous study [11] is probably mainly a consequence of a higher concentration of the tested ropivacaine (0.75%) in that study. Higher concentrated local anesthetics allowed a greater number of ropivacaine molecules to link with the nerve sheet membrane with a hastened onset and to offset the poor influence of lower concentration of the used anesthetics on the onset time [57]. The poorer realization at the beginning of anesthesia for plain 0.5% ropivacaine could be explained as clinical commonness in the gained slower onset time with a used lower concentration of ropivacaine than the usual one for third molar surgery, such as 0.75% ropivacaine [11]. Ropivacaine also has a higher dissociative constant-pKa (8.1) than the physiological values of the surrounding tissue pKa (7.4), and a lower lipid solubility and restriction of pharmacochemical ability to link with neural lipids (fat). In addition, extra neural fat cells could impede the faster onset time of local anesthesia but, paradoxically, contribute to ropivacaine to relieve enough available molecules for the linkage and breakthrough of the nerve sheet for clinically successful anesthesia, as seen in our study [58,59]. The shorter onset time of plain 0.5% bupivacaine, such as plain 0.5% ropivacaine, which does not apply to 0.5% ropivacaine and dexamethasone in this study, could be related to high bupivacaine liposolubility, which is not absolute binding dependent. Paradoxically, the high liposolubilty of local anesthetics could lead to a slower onset of anesthesia in clinical practice because high lipid solubility could contribute to sequestration in the neighboring adipose tissues and myelin sheaths, resulting in a delayed onset [57]. However, that was not observed in our study, indicating dexamethasone’s action as an adjuvant, a decisive role for favorable results. Nevertheless, the existing final dilemma in our study was the acceptable explanation for fastest/shortest onset time in the ropivacaine and dexamethasone group. Dexamethasone’s use for various purposes in medicine is well-known; it is a commonly used glucocorticoid, which is efficient as an anti-inflammatory, anti-toxicity, anti-immunity and anti-shock drug [12]. There are known reports of the beneficial effects of added dexamethasone to local anesthetics, resulting in the prolonged effectiveness of the local anesthetics, postponing the time of postoperative pain, thus impairing the use of opioid and non-opiod analgesics [60]. The pharmacokinetics of dexamethasone showed high lipohilicity, which could increase the facilitation and elicit the combination of the drug (i.e., ropivacaine) and the nerve sheath [61], which could possibly have happened in this study, enabling shorter/fastest onset time than plain 0.5% ropivacaine and plain 0.5% bupivacine.

The justifying medical reason for the perineural addition of dexamethasone to local anesthetics is also reflected through the achieved longest anesthesia and analgesia in this study. The dexamethasone + ropivacaine group evinced the statistically significant longest duration of local anesthesia (549.73 ± 224.56 min) among the ropivacaine and bupivacaine groups (277.0 ± 61.52 and 316.47 ± 151.88 min, respectively); statistically significant results of anesthesia and postoperative analgesia are in favor of ropivacaine and dexamethasone (group I), compared with group II and III. Previous scarce reports about the studied matter showed that, during third molar surgery, the duration of anesthesia with plain 0.75% ropivacaine was 412.17 ± 110.04 min [11], and these results are deficient when compared with the results of our study, where the 0.5% ropivacaine + dexamethasone group exhibited the duration of anesthesia of 549.73 ± 224.56 min. The added dexamethasone increased the lasting of intraoperative anesthesia, providing postoperative analgesia long enough and thus lessening the postoperative needs for the immediate use of opioid or non-opioid analgesics for early pain control, which is also shown in the previous studies [19]. The doubtless results in our study showed that more than one third of the studied patients absented from analgesic intake. The achieved favorable results in our study, with perineurally added dexamethasone to 0.5% ropivacain, are beneficial for the pain sequela, as well as the beneficial effects of the use of dexamethasone for postoperative lessening of all clinical sequelae (pain, trismus, swelling) in third molar surgery, well-known from previous reports [54,56,61]. The results of this study showed that, in the ropivacaine + dexamethasone group, surgery was performed with the lowest intraoperative pain and with the longest postoperative analgesia, provided by direct intra-space perineural addition of dexamethasone to long-acting 0.5% plain ropivacaine for IANB.

In the plain 0.5% ropivacaine with dexamethasone group (group I), the quality of anesthesia score (QAS) was statistically better (between the rates: successful—without pain and successful—minimal pain during the procedure without additional anesthesia after the start of surgery) compared to the plain 0.5% ropivacaine (group II) and the plain 0.5 bupivacaine (group III) QAS. It is shown by previous reports that steroids alone do not have promising results in reducing pain episodes [62,63] because there is evidence that dexamethasone administered i.v. could potentiate the patient’s reaction to pain by the suppression of β-endorphine levels; post-operative immunoreactive β-endorphin could be suppressed under even lower doses (0.1, 0.32, or 1.0 mg) of dexamethasone [64], and contrary to previous reports, there is a plethora of reports of different worldwide researchers who contributorily reported on the induced analgesia by steroids and that corticosteroids provide an analgesic effect via the anti-inflammatory or immunosuppressive mechanism [65,66]. Some researchers have found that induced analgesia by steroids is achieved through blocking neural signal transmission in nociceptive C-fibers and discontinuing the ectopic neuronal discharge [37,67], while other authors have shared the opinion that the analgesic effect of dexamethasone could be the results of modulating the normal function of potassium channels in the affected cells by pain stimuli, thus providing analgesia [68]. The intriguing, but very important, repeated question throughout this study is how to medically explain the significant contributory effects of dexamethasone in pain reduction and in providing long enough postoperative analgesia. The injected perineural dexamethasone could potentiate the synthesis of lipocortin proteins, i.e., anexin I, a molecule with a great molecule mass ≈ 37 kDa; this happens in the same cell which produces eicosanoids (prostaglandins, leucotriens, thromboxane), the substance which makes tissue pain sensitization and acts as an endogenous algesic substance; among them, prostaglandin E2 (PGE2) is the prominent member of the algesic family and contains a high nociceptive activity. Lipocortine strongly inhibits the synthesis of phospholipase A2, which is a precursor in the synthesis of prostaglandins derived from phospholipase A2. PGE2 acts through the specific receptors of adenyl cyclase (AC)/cAMP systems (cAMP-binding proteins, i.e., transcription factors, enzymes, e.g., cAMP-dependent kinases) or ion transporters, releasing substance P from the nerve endings causing pain sensation. Decreasing the level of PGE2 decreases the level of substance P and pain transmission to higher neurons in the CNS [68,69]. Glucocorticosteroids inhibit two major products of inflammation: prostaglandins and leukotrienes by blocking prostaglandin synthesis at the phospholipase A2 and cyclooxygenase/PGE isomerase (COKS1 and COKS2) level, thus acting as non-steroid anti-inflammatory drugs [70]. This could be the probable medical explanation for the beneficial effects of dexamethasone as an adjuvant on the convincing prolonged local anesthesia effects of ropivacain, thus serving as analgesics for postoperative analgesia in this study.

The dose of dexamethasone used as an adjuvant to local anesthetics for peripheral nerve block is debatable because the exact dose for that purpose has not been described yet. We choose a starting dose of 1 mL/4 mg of dexamethasone because this dose is likely to be the initial safe short-term dose (for up to 24 h) for adult patients with no adverse effects, with the same proof for this in clinical practice reported by other authors [71,72]. When added to local anesthetics, dexamethasone significantly prolongs the duration of local anesthetics, and this was also observed by Pathak et al. [73], so our similar observation about the safety and prolonged duration of anesthesia is clinically justified. It is also observed that topically applied dexamethasone is capable of producing arteriolar vasoconstriction as the result of the sensitization of the vascular muscle to noradrenalin and dexamethasone to antagonize the vasodilator effects of bradykinin and PGE [34], thus providing more intense anesthesia and analgesia.

There is a concern about the safety of the neural tissue when dexamethasone is added as an adjuvant for perineural use, but the clinician’s experience is favorable for dexamethasone used in such a way and without reported neurotoxicity caused by dexamethasone [74]. During our study, we simply did not observe any single case with any neural damage of the affected nerves with local anesthesia in combination with added dexamethasone; the precaution for a possible bad clinical dexamethasone scenario could be related to vehicle polyethylene glycol and preservative benzyl alcohol, which could be added for the production of steroids [75,76]. However, dexamethasone is produced as dexamethasone sodium phosphate, so that potential risk does not relate to our dexamethasone use. Some data suggest that dexamethasone, added as a perineural adjuvant, in fact serves as a neuroprotective drug and is widely used in clinical practice [40].

There is no consensus about the optimum route and time of administering dexamethasone intraorally for the purpose we investigated. Majid reported an improvement in the elimination of postoperative sequelae (pain included), after third molar surgery, when 4 mg of dexamethasone is given as intramuscular injection, intravenous injection, oral tablets, submucosal injection and endoalveolar powder [76]. Pedersen administered dexamethasone in the masseter muscle before the start of the operation of third molar surgery for the purpose of pain reduction [38]. Preoperatively administering 2 mL 1% ropivacaine in the pterygomandibular space for IANB anesthesia during third molar surgery and 2 mL 2% lidocaine with epinephrine 1:80.000 as a primary local anesthetic, with an additional injection of 2 mL 1% ropivacaine in the pterygomandibular space postoperatively, for the purpose of controlling the postoperative pain, showed that ropivacaine achieved greater pain reduction than lidocaine/epinephrine did and increased the duration of postoperative analgesia, whether ropivacaine was the primary or supplemental local anesthetic [77]. The suggested method of “twin–mix” mixture prepared by directly mixing 1.8 mL of 2% lignocaine with 1:200,000 epinephrine and 1 mL/4 mg dexamethasone in the syringe and administering it in the pterygomandibular space for IANB anesthesia showed similar clinical effects on the postoperative quality of life as a conventional method of injecting steroids into the intraoral-submucosal, intramuscular, intravenous and per-oral routes [78].

The used our local anesthesia approach in this study with one intra-space single injection of plain 0.5% ropivacaine with the addition of 1 mL/4 mg of dexamethasone effected a statistically significant high success rate of local anesthesia in absolute terms in 93.4% of the patients (*p* < 0.05, *p* = 0.034) and prolonged local anesthesia, providing a statistically successful postoperative analgesia for almost 11 h; other authors observed postoperative analgesia for almost 6 h when 0.75% ropivacaine was used and something more than 5 h in duration when 0.5% bupivacaine was used for IANB anesthesia [11]. Our anesthesia method resulted in one-third of the patients absenting from the postoperative intake of analgesics with the lowest mean numbers of taken analgesics, less than two analgesic tablets, amongst the tested groups. Statistically, the best quality of anesthesia score and the lowest patient’s pain intensity experience, expressed through the NRS scale, were achieved with the use of 0.5 ropivacaine and dexamethasone among all the tested groups. All these data significantly indicate that our method provides successful and profound IANB anesthesia in a simple and easy way, long enough to achieve successful postoperative analgesia, and it is efficient and applicable in every day clinical practice.

Our observations and recorded data show that the measured secondary anesthetic outcomes (adverse reactions) of the tested anesthetic solutions are seldom noticed and insignificant (6.7%) in almost all groups during this investigation and that they are common in ambulatory surgery practice when local anesthetics are used for IANB anesthesia [51]. Kaufman et al. [79] found an incidence of 26.2% of patients who experienced different adverse reactions receiving local anesthetics for different dental procedures and oral and maxillofacial ambulatory surgery; among all adverse reactions, dizziness happens in 4.4% of the subjects who receive a local anesthetic injection. Out of all patients, 45% percent of the patients experience adverse reactions at the time of injection and 29% within 2 h post injection. In our study, short-term dizziness occurred randomly in 4.4% of patients in groups I and III, such as in other studies [79]. Dizziness separately occurred in 1 patient in groups I and III, up to 5 min post injection of ropivacaine with dexamethasone and bupivacaine, with a subsequent quick recovery after 5–10 min post injection and without impact on the general health status and further course of surgery. Positive aspiration occurred in group I (one patient/6.7%) and group II (two patients/13.3%). Roberstone [80] published an incidence of 15–17% of positive aspiration during the Gow-Gates techniques, while Donkor et al. [81] noticed 22% of positive aspiration during the Halsteade technique for IANB; those data were found in 14 studies cited by Watson and Gow-Gates [82], so our observation is not a surprise. Hematoma occurred in 2 (4.4%) of patients, reported separately in groups II and III, but this adverse reaction is insignificant because it was reported in previous studies in a much higher percent (22%) [81,82,83]. All the recorded adverse CNS reactions may happen in clinical practice when dealing with the use of local anesthetics, and similar side-effects are recorded when using bupivacaine and other local anesthetics for nerve blocks [84,85]. Some of them (dizziness, drowsiness) are related to several factors: the route of administration of local anesthetics, the chosen anesthetics and the physical status of the patient, and there are results of depression of the CNS due to the toxic reaction of the injected drug [85,86]. Some speculative explanation for the adverse reaction of the given anesthetic (possible for all anesthetics) could be related to the molecular neurotoxic effects of local anesthetics, caused by the increased toxic concentration of alcohol in the neuron, produced during hydrolysis of ester or amide-type of local anesthetics [87]. Recorded toxic reactions presented in this study occurred as rare cases and as a transient manifestation and without any medical consequence for the patients. Transient paresthesia of the lingual nerve happened in 1 (2.2%) of all the patients in our study but not in another study [88], and we could not relate it to the given local anesthetics in this study but rather to the given technique of local anesthesia [87,88,89]. However, it should be pointed out that some local anesthetics, such as Articaine 4% and Prilocaine 3–4%, can cause toxic effects on the alveolar inferior or lingual nerves, with sensory disturbance [89], due to the high concentration of anesthetics [90]; Articaine induced paresthesia could reach 71% of the given anesthesia [89]. Our reported case with lingual paresthesia had a nerve recovery within three post-operative weeks, so we assume that the patient’s experienced sensory disturbance was related to neural injury, such as neurapraxia [91], because it lasted three weeks and ended with the complete resolution of lingual sensory. In addition, this could have happened during third molar surgery [91] or during local anesthesia administration [88]. The prolonged lingual paresthesia is also seen in dental and wisdom tooth surgery clinical practice and is reported in the literature [92].

In this study, the achieved beneficiary effects on local anesthesia outcomes by means of plain 0.5% ropivacaine with perineurally added dexamethasone are also found by other authors [93].

### Strengths and Weaknesses of the Study

The limitations of this study are related to the mean age range of the included patients for this investigation; in all three groups of patients, the mean age range was up to the third decade of life (younger population), so we did not obtain scientific data about the older population and their response to the given anesthetic solutions. Furthermore, the patients with chronic systemic diseases were excluded from this study for safety reasons. On the other hand, those groups could be of particular interest for the response to given anesthetic solutions, such as the used ones in this study, and further investigation is necessary to evaluate this group of patients. The investigated cohort in this study is limited to 15 subjects per group. Even though all the collected data are processed thorough valid statistical methods, the obtained results are statistically significant and clinically valuable in everyday practice.

## 5. Conclusions

The present study showed that plain 0.5% ropivacaine, in combination with 4 mg dexamethasone, administered in one intra-space injection into the pterygomandibular space, gained a prolonged and profound long-lasting inferior alveolar nerve block anesthesia for third molar surgical extractions. Postoperatively, analgesia lasts 11 h in duration, with an efficacy of 93.4% of given anesthesia. The used ropivacaine and dexamethasone anesthetic solution in this study usefully served as an analgesic in providing successful postoperative analgesia, with more than one-third of the operated patients absented from analgesics intake, where pain reduction is usually provided by opioid or non-opioid analgesics.

## Figures and Tables

**Table 1:** Demographics of enrolled patients in the study.

	Rop + Dex (Group I) *N* = 15		Rop (Group II) *N* = 15		Bup (Group III) *N* = 15	
Age	29.07 ± 7.40		24.53 ± 6.22		27.58 ± 10.32	
95% CI of mean age	24.97–33.16		21.09–27.98		21.86–33.29	
Sex						
Male	7	46.7 (%)	3	20.0 (%)	5	33.3 (%)
Female	8	53.3 (%)	12	80.0 (%)	10	66.7 (%)

Note: The mean age for all tested groups is 27.06 ± 8.20. Abbreviations: rop + dex (plain 0.5% ropivacaine plus 1 mL dexamethasone), rop (plain 0.5% ropivacaine) and bup (0.5% plain bupivacaine).

**Table 2:** Collected parameters for separated primary local anesthesia outcomes and observed secondary outcomes.

	Ropivacaine + Dex *N* = 15	Ropivacaine *N* = 15	Bupivacaine *N* = 15	*p*^1^ Values
Primary local anesthesia outcomes				
Duration of anesthesia (min)	549.73 ± 224.56 ^b^	277.0 ± 61.52	316.47 ± 151.88 ^a^	0.001
Secondary local anesthesia outcomes				
Onset time of anesthesia (min)	4.13 ± 1.30	5.40 ± 2.10	4.47 ± 1.46	0.289
Success of local anesthesia in absolute terms #/%, with additional anesthesia #/%	14 (93.4%) 1 (6.66%)	10 (66.7%) 5 (33.33%)	9 (60%) 6 (40%)	0.034 ^2^
Amount of anesthesia per patient (mL)	4.13	4.66	4.8	0.097
The # of patients used analgesics #/%	10 (66.7%)	15 (100%/0%)	13 (86.7%)	0.052
NRS of pain when the first anlgesics used	3.70 ± 0.68	4.93 ± 0.96	4.50 ± 0.76	0.004
Duration of analgesia when analgesics used for the first time (min)	654.9 ± 198.4 ^b^	345.4 ± 88.0	413.7 ± 152.3	0.001
# of analgesics during 24 h postoeratively	1.90 ± 0.57 ^b^	2.80 ± 0.86	2.36 ± 0.84	0.027
Dizziness #/%	1 (2.22%)	0 (0.0%)	1 (2.22%)	1.000 ^3^
Drowiness #/%	0 (0.0%)	0 (0.0%)	1 (2.22%)	1.000 ^3^
Haematoma #/%	0 (0.0%)	1 (2.22%)	1 (2.22%)	1.000 ^3^
Positive aspiration #/%	1 (2.22%)	0 (0.0%)	0 (0.0%)	1.000 ^3^
Transient paresthesia of n.lingualis #/%	0 (0.0%)	0 (0.0%)	1 (2.22%)	1.000 ^3^

^1^ Kruskal–Wallis test, ^2^ Chi-squared test, ^3^ Fisher test, ^a^ *p* < 0.05 vs ROP + Dex, ^b^ *p* < 0.05 vs. Ropivakain. Abbreviation: Dex(dexamethasone).

**Table 3:** Achieved quality of anesthesia scores (QAS).

Administered Local Anesthesia Solutions	Achieved Mark								Mean Quality of Achieved Anesthesia	*p* Value ^1^
	1	2	3	4	5	6	7	8		
GROUP I 0.5% Ropivacaine and 4 mg Dex	9	5	1	0	0	0	0	0	1.47 ± 0.38	0.037
GROUP II Plain 0.5% Ropivacaine	3	7	2	2	1	0	0	0	2.40 ± 1.31 ^a^	
GROUP III 0.5% Bupivacaine	4	5	4	2	0	0	0	0	2.27 ± 1.00 ^a^	

^1^ Kruskal–Wallis test, ^a^ vs Ropivacaine plus dex, *p* < 0.05; Abbreviations: dex (dexamethasone). Meaning of scores: 1—successful, without pain. 2—successful, minimal pain during procedure without additional anesthesia after the start of surgery. 3—successful, minimal pain after first anesthesia, no pain after another anesthesia. 4—limited success, pain during surgery after first anesthesia and after second anesthesia, but surgery completed without third anesthesia. 5—limited success, pain during surgery begins after two anesthesia but surgery completed without third anesthesia. 6—failure, pain during surgery after first anesthesia, pain during surgery after second anesthesia, needed third anesthesia. 7—failure, pain during surgery begins immediately after two anesthesia required third anesthesia. 8—failure/no anesthesia achieved after two local anesthesia administration, required third anesthesia or surgery postponed.

## Data Availability

Not applicable.
